# Teamarbeit und Stress bei Routineeingriffen: eine Beobachtungsstudie multiprofessioneller OP-Teams

**DOI:** 10.1007/s00113-021-00977-w

**Published:** 2021-03-05

**Authors:** Stefanie Passauer-Baierl, Ulla Stumpf, Matthias Weigl

**Affiliations:** 1 Institut und Poliklinik für Arbeits‑, Sozial- und Umweltmedizin, Klinikum der Ludwig-Maximilians-Universität München, München, Deutschland; 2 Beratung und Training, Human Factors und Patientensicherheit, Parkstetten, Deutschland; 3 Klinik für Allgemeine, Unfall- und Wiederherstellungschirurgie, Klinikum der Ludwig-Maximilians-Universität München, München, Deutschland; 4 Institut für Patientensicherheit, Universitätsklinikum Bonn, Bonn, Deutschland

**Keywords:** Kommunikation, Interdisziplinäre Zusammenarbeit, OTAS, Befragung, Patientensicherheit, Communication, Interdisciplinary teamwork, OTAS, Questionnaire, Patient safety

## Abstract

**Hintergrund:**

Effektive interprofessionelle Teamarbeit im Operationssaal (OP) und intraoperativer Stress sind von großer Bedeutung für Patientensicherheit und Versorgungsqualität. Dennoch gibt es nur wenige systematische Studien zum Zusammenhang von Teamarbeit im OP und Arbeitsstress.

**Ziele der Arbeit:**

Untersuchung des Zusammenhangs von Teamarbeit und empfundenem Stress bei Routineeingriffen – für das OP-Team als Gesamtheit sowie für die einzelnen Professionen Chirurgie, Anästhesie und Pflege.

**Material und Methoden:**

Durchgeführt wurde eine Mehrmethodenstudie bestehend aus Expertenbeobachtungen mittels eines standardisierten Beobachtungsinstruments (OTAS-D) und systematischer Selbstberichte des gesamten OP-Teams. Erfasst wurden 64 elektive Routineeingriffe unterschiedlicher chirurgischer Fachbereiche. Die statistischen Zusammenhangsanalysen unter Kontrolle prozeduraler Einflussfaktoren wurden mit „Mixed-effects“-Regressionsmodellen berechnet.

**Ergebnisse:**

Die Güte der intraoperativen Teamarbeit lag auf mittlerem Niveau. Der situative Stress während des Eingriffs wurde durch die Befragten eher auf niedrigerem Niveau berichtet, mit signifikanten Unterschieden zwischen den Professionen Chirurgie, Pflege und Anästhesie. Mitglieder des chirurgischen Teams berichteten im Durchschnitt die höchsten Stressniveaus. Ein genereller Zusammenhang zwischen Teamarbeit und Stresserleben konnte nicht beobachtet werden, allerdings für die einzelnen Professionen: Für das chirurgische Team ergaben sich signifikante, positive Zusammenhänge, sowie für die Teamarbeitsdimensionen Zusammenarbeit und Führung. Signifikante negative Zusammenhänge ergaben sich für das Pflegeteam hinsichtlich der Qualität der interdisziplinären Teamarbeit insgesamt sowie für die Teamarbeitsdimension Team-Monitoring.

**Diskussion:**

Die Effekte interprofessioneller Zusammenarbeit im OP auf subjektives Stressempfinden bei Routineeingriffen hängen von Professionszugehörigkeit, Aufgabe und Tätigkeit ab. Weitere Forschungsarbeit ist notwendig, inwiefern gute Teamarbeit bei Routineeingriffen innerhalb und über die Professionen hinweg intraoperativen Stress beeinflusst.

**Zusatzmaterial online:**

Die Online-Version dieses Beitrags (10.1007/s00113-021-00977-w) enthält eine vollständige Liste mit Kurzbeschreibungen der beobachteten Eingriffe. Beitrag und Zusatzmaterial stehen Ihnen auf www.springermedizin.de/link/10.1007/s00113-021-00977-w zur Verfügung. 
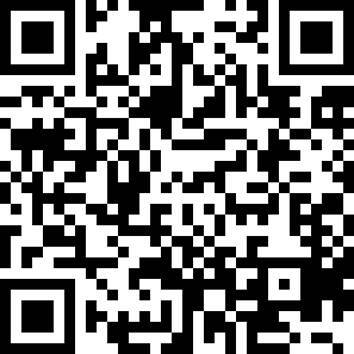

## Abstract (en)

**Background:**

Effective interprofessional teamwork and stress are important factors for quality of care and patient safety in the operating room (OR); however, there are just a few systematic investigations into the relationship of OR teamwork and occupational stress.

**Objective:**

Determination of the relationship between interdisciplinary OR teamwork and stress in routine procedures, for the whole OR team as well as individual professions.

**Methods:**

Multimethod study with expert observations using a standardized observation tool (OTAS-D) and systematic self-reports of the entire OR team. A total of 64 elective interventions across different surgical departments were observed. Relationships were calculated using mixed-effects regression models with control of procedural and provider characteristics.

**Results:**

The quality of the intraoperative teamwork was at a medium level. Reported stress during interventions was at comparatively low levels, with significant differences between the professions of surgery, nursing and anesthesiology. Members of the surgical team reported the highest stress levels. An association between teamwork and perceived stress could not be determined for the entire OR team; however, within the surgical sub-team there were significant positive correlations for the quality of teamwork and stress (as well as for the teamwork dimensions of collaboration and leadership). For the nursing sub-team, we observed significant negative correlations with overall teamwork as well as with the dimension team monitoring.

**Conclusion:**

The findings suggest a profession-specific relationship between OR teamwork and occupational stress. Further research is necessary to investigate to what extent successful OR teamwork during routine procedures contributes to intraoperative stress.

## Hintergrund

Effektive Teamarbeit und Stress gehören zu den bedeutendsten Faktoren im OP, da sie die operative Leistung, Patientenergebnisse und -sicherheit beeinflussen können. Über den Zusammenhang dieser Faktoren ist allerdings noch wenig bekannt, insbesondere im Hinblick auf professionsübergreifende Zusammenarbeit sowie elektive Routineeingriffe.

Die Qualität der Zusammenarbeit verschiedener Professionen und Berufsgruppen im OP hat nicht nur Einfluss auf die klinische Versorgung [26]; mangelhafte interprofessionelle Zusammenarbeit ist auch einer der häufigsten bedingenden Faktoren kritischer Vorfälle im OP [1, 29]. Fehler in der Zusammenarbeit erhöhen die Wahrscheinlichkeit von Komplikationen und Todesfällen [18]. Ursachen für unerwünschte Ereignisse (engl.: „adverse events“) sind häufig auf Kommunikationsprobleme oder ineffiziente Teamarbeit zurückzuführen [17]. Gleichzeitig ist effektive Teamarbeit auch Grundlage der Fehlervermeidung und -reduzierung [25]. Unter effektiver interprofessioneller Teamarbeit ist der dynamische, möglichst reibungslose Austausch von Informationen und Ressourcen zwischen allen Teammitgliedern zu verstehen. Hierzu gehören auch gute Kommunikation, Management und Timing von Aktivitäten und Aufgaben, unterstützendes Verhalten, Führungsverhalten sowie Aufmerksamkeit der Teammitglieder für laufende Vorgänge [12, 22].

Intraoperativer Stress des Personals, definiert als körperliche und geistige Belastungsreaktion, ist ein weiterer kritischer Faktor – nicht nur bei besonders herausfordernden Eingriffen oder auftretenden Komplikationen. Auch bei Routineeingriffen sind Ärzte und Pflegekräfte häufig intraoperativen Stressoren wie Ablenkungen, Zeitdruck, Komplexität des Eingriffs, Blutverlust oder spezifischen Patientenanforderungen ausgesetzt [4, 7, 28, 33]. Die Reaktion auf Stressoren sowie der Umgang mit Stress sind dabei individuell und subjektiv: Ob und in welchem Umfang ein Stressor eine Stressreaktion auslöst, hängt von der subjektiven Bewertung ab [16]. Darüber hinaus bestehen individuelle Unterschiede, ab welchem Niveau Stress als unangenehm empfunden wird.

Stressreaktionen bzw. inadäquater Umgang mit den Stresssituationen können die chirurgische Leistung negativ beeinflussen [32, 35] und beeinträchtigen die sog. nichttechnischen Fähigkeiten wie Koordination, Kommunikation und intraoperative Entscheidungen [4, 7, 30]. Zudem gibt es auch erste Hinweise, dass Stress, die Zusammenarbeit sowie die Kommunikation des OP-Teams im Zusammenhang stehen [2, 4, 6, 28]. Allerdings ist die bestehende Evidenz zum Zusammenhang interprofessioneller Teamarbeit im OP und Stress limitiert, insbesondere für Routineeingriffe [6]. Da jedoch elektive, mehrheitlich unkritische Eingriffe für das Gros des Personals alltäglich sind, sind systematische Studien zu Entstehung und Auswirkung von Stress und OP-Teamarbeit im Routinebetrieb und unter Berücksichtigung aller bzw. mehrerer Fachrichtungen notwendig.

Ziel dieser Untersuchung war es, Zusammenhänge von interprofessioneller Teamarbeit im OP und subjektiv empfundenem Stress für das gesamte OP-Team im Routinebetrieb unter Berücksichtigung weiterer einflussnehmender Faktoren zu beschreiben.

## Fragestellungen und Hypothesen der Studie

Auf Basis einer kombinierten Beobachtungs- und Befragungsstudie wurde der Frage nachgegangen, ob ein Zusammenhang zwischen der Güte der interprofessionellen Teamarbeit und des berichteten Stresses des OP-Teams beobachtbar ist. Auf Basis der Hypothese, dass Zusammenhänge zwischen defizitärer Zusammenarbeit der Teammitglieder und ihrem subjektiven Stress bestehen, lauteten die spezifischen Einzelfragen:

1. Wie ist die Ausprägung der interprofessionellen Teamarbeit und des Stresserlebens in der untersuchten Gruppe insgesamt und innerhalb der OP-Professionen?
2. Welche individuellen, prozeduralen oder patientenspezifischen Merkmale weisen ebenfalls einen Zusammenhang zum berichteten Stresserleben auf?
3. Ist ein Zusammenhang zwischen einzelnen Dimensionen der interprofessionellen Teamarbeit und dem subjektiv berichteten Stresserleben der Teammitglieder des OP-Personals (Teilfragestellung 3a), zwischen der Teamarbeit und dem Stresserleben pro Profession (3b) sowie zwischen den Dimensionen der OP-Teamarbeit und dem Stresserleben innerhalb der Professionen beobachtbar (3c)?

## Methodik

### Studiendesign und klinisches Setting

Um der Komplexität des Themas gerecht zu werden, wurde ein Mehrmethodendesign von Expertenbeobachtungen und standardisierten Selbstberichten eingesetzt. Alle Erhebungen wurden in Operationssälen zweier Standorte eines süddeutschen Universitätsklinikums der Maximalversorgungsstufe durchgeführt. Die Operationssäle waren vergleichbar, in Hinsicht auf Größe, Ausstattung, Arbeitsorganisation und personelle Besetzung.

Die Studie wurde vorab durch die zuständige Ethikkommission der medizinischen Fakultät positiv beurteilt (Nr. 539-11). Auch die Personalvertretung stimmte dem Studienprotokoll zu. Um rechtzeitig das Einverständnis des Personals einzuholen, wurden alle Mitglieder der Teams vorab über die Durchführung der Studie in den abteilungsinternen Morgenbesprechungen und im Intranet informiert. Alle Untersuchungsschritte waren freiwillig. Jedes Mitglied des OP-Teams hatte jederzeit die Möglichkeit, die Beobachtung zu untersagen. In der gesamten Erhebung wurden keine Patientenidentitäten erfasst.

### Untersuchungsgruppe und Einschlusskriterien

Um dem Ziel einer ersten Analyse der Zusammenhänge von Zusammenarbeit und Stress mit generalisierbaren Ergebnissen Rechnung zu tragen, wurde eine Gelegenheitsstichprobe elektiver Eingriffe rekrutiert (detaillierte Auflistung und Beschreibung der Eingriffe: Zusatzmaterial online). Alle Beobachtungen wurden tagsüber im OP-Routine-Betrieb durchgeführt. Eingeschlossen wurden alle für den Beobachtungstag gelisteten Eingriffe verschiedener chirurgischer Disziplinen (Viszeralchirurgie, Orthopädie/Unfallchirurgie, Gefäßchirurgie, plastische/Handchirurgie, Herzchirurgie sowie Thoraxchirurgie). Aufgrund der Konzentration auf Teamarbeit und Stressempfinden im Routinebetrieb wurden Akuteingriffe und Notoperationen ausgeschlossen; ebenso wie Eingriffe mit einer geschätzten Dauer von über 4 h (da mit höherer Dauer die Personalwechsel im Anästhesie- und Pflegeteam zunehmen sowie die Validität und Reliabilität der teilnehmenden Beobachtung nicht gewährleistet ist). Nachträglich wurden 3 Eingriffe ausgeschlossen, die weniger als 20 min dauerten. Die Besetzung der OP-Teams wechselte über die eingeschlossenen Eingriffe hinweg.

### Ablauf der Datenerhebung

Durchgeführt wurden die teilnehmenden OP-Beobachtungen zur Bewertung der interprofessionellen Teamarbeit von jeweils einem/einer im Umgang mit dem Beobachtungsinstrument trainierten Beobachter/-in (SPB, MW). Deren Beobachterübereinstimmung wurde im Vorfeld durch eine Reliabilitätsstudie geprüft und bestätigt [23].

Der Fragebogen zum intraoperativen Stress des Personals wurde im Anschluss an den Eingriff, wenn der/die Patient*in den OP verlassen hatte (bei Chirurgen und OP-Pflegekräften) oder im Anschluss an die postoperative Versorgung und Übergabe (Anästhesie), verteilt und zeitnah wieder eingesammelt.

### Erhebungsinstrumente

#### Interprofessionelle Teamarbeit („Observational Teamwork Assessment for Surgery“)

Die Güte der OP-Teamarbeit wurde mithilfe der deutschen Version des Beobachtungsinstruments „Observational Teamwork Assessment for Surgery“ (OTAS-D) beurteilt. Das OTAS‑D ist ein international etabliertes, standardisiertes, reliables und valides Beobachtungsinstrument [19, 23, 31]. Die Bewertung erfolgte mittels einer teilnehmenden Beobachtung während der gesamten Operation unter Einschluss aller OP-Phasen und vornehmlich am Eingriff beteiligten Professionen. OTAS‑D ermöglicht eine Beurteilung der Teamarbeit des gesamten Teams sowie innerhalb der einzelnen OP-Professionen. Diese werden im Folgenden als Subteams bezeichnet: das chirurgische Subteam, bestehend aus operierendem Chirurgen und Assistenten, das pflegerische Subteam, bestehend aus instrumentierender Pflegekraft, Springer und ggf. Lagerungspflegekräften sowie das anästhesiologische Subteam mit Anästhesisten und Anästhesiepflege [22, 23, 31].

Berichtet werden im Folgenden die Bewertungen aus der intraoperativen Phase (Zeitraum vom Schnitt bzw. Zugang zum Zielorgan bis Nahtvollendung). Die Teamarbeit wird anhand von 5 Verhaltensdimensionen eingestuft, die als maßgeblich für sichere und effektive Patientenversorgung im OP gelten [12, 22]:

1. Kommunikation (Qualität und Quantität der Informationen, die unter den Teammitgliedern ausgetauscht werden),
2. Koordination (Management und Timing von Aktivitäten und Aufgaben),
3. Zusammenarbeit/unterstützendes Verhalten (Unterstützung und Hilfe von Teammitgliedern, Unterstützung anderer und Korrektur von Fehlern),
4. Führung (Bestimmung von Richtungen, Durchsetzungsvermögen und Unterstützung unter den Teammitgliedern),
5. Team-Monitoring/situatives Bewusstsein (Teambeobachtung und Aufmerksamkeit für laufende Vorgänge).

Die Bewertung der Teamleistung erfolgt mittels einer 7‑stufigen Ordinalskala (0–6), wobei der Skalenmittelpunkt (3) eine durchschnittliche Leistung in einer Verhaltensdimension widerspiegelt. Die höchste Wertung (6) zeigt eine signifikante Verbesserung durch beispielhaftes Verhalten an, während die niedrigste Wertung (0) eine schwere Beeinträchtigung der Zusammenarbeit ausdrückt.

#### Befragung zum intraoperativen Stress

Alle Mitglieder des beobachteten OP-Teams wurden um eine retrospektive Einschätzung ihres subjektiven Stresserlebens während des beobachteten Eingriffs gebeten. Im Anschluss an die Operation wurde *in einem durch die Teammitglieder auszufüllenden Bogen *die Frage gestellt: „Wie angespannt waren Sie während der Operation?“ Diese wurde mittels einer visuellen Skala (0: nicht angespannt; 100: sehr angespannt) beantwortet. Die Frage entstammt dem NASA Task Load Index, einem etablierten und validierten Verfahren [9, 10].

#### Prozedurale, patientenspezifische und individuelle Einflussmerkmale

Erhoben wurden zusätzlich mehrere prozedurale, patientenspezifische und individuelle Merkmale, die gemäß unserer Hypothese evtl. Einfluss auf das Stresserleben nehmen. Erfasst wurden die Dauer der intraoperativen Phase (Zeit von Schnitt bis Naht), die Art des Eingriffs bzw. operierender Fachbereich, ob es sich um einen minimal-invasiv oder offen durchgeführten Eingriff handelte, und ob eine Operation unter Anleitung (Assistenzarzt/-ärztin als Hauptoperateur/-in unter Anleitung einer/eines Vorgesetzten) durchgeführt wurde. Als Freitext konnten die während des Eingriffs anwesenden Personen sowie besondere Vorkommnisse festgehalten werden. Zudem wurde der ASA-Score des Patienten erfasst. Im Fragebogen lieferten die OP-Team-Mitglieder Angaben zu ihrer Berufserfahrung (<5 Jahre, 5 bis 10 Jahre, > 10 Jahre) und ihrer Profession (Pflege, Chirurgie, Anästhesie).

### Statistische Analysen

Tests auf Gruppenunterschiede wurden mittels Varianzanalyse und t‑Tests durchgeführt. Zur Klärung der Hauptfragestellung zum Zusammenhang zwischen der OP-Teamarbeit und Stress der Teammitglieder sowie der Teilfragestellung 3a wurde ein „Mixed-effects“-Regressionsmodell [24] erstellt. Dieses umfasste als Prädiktoren die *Bewertungen zur Teamarbeit des OP-Teams sowie die einzelnen Teamarbeitsdimensionen unter Kontrolle *prozeduraler Einflussfaktoren *(Dauer der Operation, ASA-Score des Patienten, operierender Fachbereich) sowie als Endpunkt den berichteten intraoperativen Stress*. Analyseeinheit waren die durchgeführten Operationen, in denen die Einzelberichte des Teams modelliert wurden. Die Fragestellungen 3b) sowie 3c) wurden mittels einer multiplen Regressionsanalyse untersucht. Alle statistischen Analysen wurden mit IBM SPSS 25 (IBM Inc., Chicago) durchgeführt.

## Ergebnisse

### Beschreibung der beobachteten Eingriffe

Insgesamt wurden 64 Routineeingriffe ausgewertet. Die Beobachtungen dauerten durchschnittlich 157,81 min (SD: 64,41; Min.: 55 min; Max.: 354). Die Eingriffe selbst, gemessen vom Schnitt bis zum Verschluss, dauerten im Mittel 87,89 min (SD: 52,53; Min.: 20; Max.: 237). Insgesamt wurden 5625 min (93,75 h) intraoperativer Teamarbeit bewertet. Am häufigsten wurden Eingriffe in der Viszeralchirurgie beobachtet (*n* = 33; 51,6 %), gefolgt von orthopädischen/unfallchirurgischen Eingriffen (*n* = 20; 31,3 %). Erfasst wurden auch 5 Eingriffe der Gefäßchirurgie (7,8 %), 3 der plastischen/Handchirurgie (4,7 %) sowie jeweils einer der Fachbereiche Herzchirurgie, Thoraxchirurgie und Sonstiges (VAC-Wechsel mit Materialentfernung). Von den 64 beobachteten Eingriffen wurden 22 (34,4 %) minimal-invasiv durchgeführt. 10 Eingriffe wurden von Assistenten unter Supervision durchgeführt.

Der mittlere ASA-Score der Patienten lag bei M = 2,3 (SD: 0,71; Min.: 1; Max.: 4). 9,4 % der Patienten wiesen einen ASA-Score von 1, 56,3 % einen ASA-Score von 2, 29,7 % einen Score von 3 und 4,7 % einen ASA-Score von 4 auf.

Zusätzlich wurden 265 Fragebogen *zum intraoperativen Stresserleben* durch das OP-Personal ausgefüllt, mit durchschnittlich 4,38 Fragebogen pro Eingriff (SD: 0,97; Min.: 2; Max. 6). Die Beteiligungsquote lag bei 62,5 %. 95 Fragebogen (35,8 %) wurden von OP-Pflegekräften ausgefüllt, 107 (40,4 %) durch das chirurgische Subteam und 63 (23,8 %) von Vertretern des anästhesiologischen Teams. 42,6 % der Befragten weisen eine Berufserfahrung von über 10 Jahren, 23,8 % von 5 bis 10 Jahren und 32,8 % von unter 5 Jahren auf (keine Angabe bei 2 Fragebogen).

### Güte der intraoperativen Teamarbeit (Fragestellung 1)

Die mittlere Wertung der interprofessionellen Teamarbeit über alle 5 Verhaltensdimensionen und Subteams hinweg betrug M = 3,38 (SD: 0,36), *liegt also im mittleren Bereich der Skala*. Die jeweiligen Werte für die einzelnen OP-Professionen betrugen für das chirurgische Team M = 3,54 (SD: 0,73), für das Pflegeteam M = 3,33 (SD: 0,57) sowie M = 3,26 (SD: 0,50) für das Anästhesieteam.

Die jeweiligen Mittelwerte für die 5 Verhaltensdimensionen des Gesamtteams sowie der Subteams finden sich in Tab. 1. Weder für die Gesamtwertung noch für die einzelnen OTAS-Dimensionen ergaben sich signifikante Mittelwertunterschiede zwischen den Berufsgruppen (Ergebnisse dieser Unterschiedsprüfung auf Anfrage verfügbar).

**Table 1:** 

OTAS‑D, Verhaltens-dimension	OTAS‑D, Mittelwerte (und Standardabweichung)			
	Gesamtes Team	Chirurgisches Team	Pflegeteam	Anästhesieteam
Kommunikation	3,44 (0,55)	3,59 (1,17)	3,34 (0,74)	3,38 (0,86)
Koordination	3,41 (0,46)	3,64 (0,76)	3,34 (0,91)	3,25 (0,47)
Zusammenarbeit	3,43 (0,49)	3,58 (0,85)	3,31 (0,66)	3,39 (0,61)
Führung	3,38 (0,35)	3,80 (0,84)	3,27 (0,65)	3,06 (0,50)
Team-Monitoring	3,23 (0,55)	3,11 (0,89)	3,38 (0,85)	3,22 (1,15)

*n* = 64 Eingriffe, Skala OTAS-D: 0, völlig unzureichend; bis 6, sehr gute Teamarbeit

### Intraoperatives Stresserleben

Im Mittel gab das befragte OP-Personal den intraoperativen Stress mit M = 23,96 an (SD: 19,05; *n* = 264); die Pflegekräfte mit M = 20,68 (SD: 18,24; *n* = 95), das chirurgische Subteam mit M = 27,75 (SD: 19,14; *n* = 106) und die Anästhesie mit M = 22,52 (SD: 19,29; *n* = 63). Dieser Unterschied im berichteten Stress der Professionen war signifikant (F(2,261) = 3,76, *p* < 0,05).

### Einflussfaktoren für das intraoperative Stresserleben (Fragestellung 2)

Keine statistisch signifikanten Unterschiede für das Stresserleben ergaben sich für die prozeduralen Merkmale minimal-invasiv vs. offen durchgeführte Eingriffe, ob die Operation unter Anleitung durchgeführt wurde, sowie hinsichtlich der Größe des OP-Teams. Die Dauer des Eingriffs und der intraoperative Stress des gesamten OP-Teams korrelierten positiv miteinander (r = 0,16; *p* < 0,01). Bei der getrennten Betrachtung der Subteams ergaben sich hier für Pflege und Anästhesie keine Zusammenhänge; ein signifikanter Zusammenhang zwischen dem Stresserleben und der Operationsdauer ergab sich für das chirurgische Subteam: r = 0,24 (*p* < 0,05). Bezüglich des individuellen Faktors Berufserfahrung des Personals wies die Varianzanalyse keine signifikanten Unterschiede im Stresserleben auf. Der patientenbezogene Faktor des ASA-Scores korrelierte lediglich mit dem Stresserleben des anästhesiologischen Subteams (r = 0,33; *p* < 0,001).

### Zusammenhang zwischen interprofessioneller Teamarbeit und Stress (Fragestellung 3)

Unsere Hauptfragestellung betraf den Zusammenhang der interprofessionellen Teamarbeit sowie des erlebten intraoperativen Stresses des OP-Teams. In unserem multivariaten Modell wurden gemäß den Ergebnissen aus Fragestellung 2 als Kontrollvariablen die Dauer der Operation, der operierende Fachbereich, der ASA-Score sowie die Berufsgruppen berücksichtigt. Für den Zusammenhang der Güte der Teamarbeit und des Stresserlebens aller Teammitglieder ergab sich kein signifikantes Ergebnis (Tab. 2).

**Table 2:** 

Prädiktor	Schätzer	95 % KI	Standardfehler	Signifikanz
OP-Teamarbeit, allgemein	−3,75	−10,42; 2,92	3,33	0,27
*Teamarbeitsdimensionen*				
Kommunikation	−1,64	−6,15; 2,87	2,26	0,47
Koordination	−3,31	−8,43; 1,81	2,56	0,20
Zusammenarbeit	0,27	−4,76; 5,31	2,52	0,91
Führung	0,62	−6,28; 7,52	3,45	0,86

„Multilevel-Model“-Parameterschätzungen, adjustiert für Dauer der Operation, ASA-Score und Art des Eingriffs (operierender Fachbereich), *KI* Konfidenzintervall

Auch für die einzelnen Teamarbeitsdimensionen Kommunikation, Koordination, Zusammenarbeit und Führung ergaben sich im jeweiligen Modell keine signifikanten Zusammenhänge (Fragestellung 3a; Tab. 2). Für die Dimension Team-Monitoring konnte aufgrund zu geringer Intraklassenvariabilität trotz Erfüllung der Konvergenzkriterien keine gültige Multilevelanalyse durchgeführt werden. Die Analyse erfolgte aus diesem Grund über eine lineare Regressionsanalyse (mit einem pro Eingriff gemittelten Stresswert als Outcome): β = 0,05, 95%-KI [−3,73; 8,76]; *n* = 265.

### Teamarbeit-Stress-Zusammenhänge pro Profession (Fragestellungen 3b und 3c)

In einem zusätzlichen Auswertungsschritt untersuchten wir Zusammenhänge zwischen der interprofessionellen Teamarbeit sowie des berichteten Stresses, getrennt nach Subteams. Die Ergebnisse zeigt Tab. 3: Für das chirurgische Subteam ergaben sich signifikante Zusammenhänge bei der Teamarbeit insgesamt sowie für die Dimensionen Zusammenarbeit und Führung. Für die OP-Pflege identifizierten wir signifikante Zusammenhänge für die Teamarbeit insgesamt sowie für die Dimension Team-Monitoring. Für die Anästhesie ergaben sich keine signifikanten Zusammenhänge (Tab. 3).

**Table 3:** 

Subteam	OTAS‑D	B	Signifikanz	95 % KI
Chirurgie	Teamarbeit, insgesamt	0,30	**0,02**	1,27; 11,76
	Kommunikation	0,14	0,29	−3,40; 11,26
	Koordination	0,07	0,57	−6,19; 11,19
	Zusammenarbeit	0,33	**0,01**	2,79; 18,64
	Führung	0,26	**0,04**	0,86; 23,49
	Team-Monitoring	0,04	0,78	−8,52; 6,42
Pflege	Teamarbeit, insgesamt	−0,27	**0,04**	−13,93; −0,37
	Kommunikation	−0,12	0,37	−10,53; 3,99
	Koordination	−0,25	0,05	−16,86; 0,03
	Zusammenarbeit	−0,13	0,35	−12,03; 4,32
	Führung	−0,12	0,36	−16,10; 6,00
	Team-Monitoring	−0,28	**0,04**	−15,88; −0,62
Anästhesie	Teamarbeit, insgesamt	0,01	0,93	−10,01; 10,96
	Kommunikation	−0,11	0,42	−13,53; 5,73
	Koordination	−0,01	0,95	−11,52; 10,81
	Zusammenarbeit	−0,12	0,20	−17,54; 3,74
	Führung	−0,08	0,57	−18,19; 10,15
	Team-Monitoring	−0,12	0,39	−14,80; 5,84

Anmerkungen: adjustiert für Dauer der Operation, ASA-Score und operierenden Fachbereich; *B* Regressionskoeffizient; *KI* Konfidenzintervall; fett: *p* < 0,05

## Diskussion

Gute und effektive Teamarbeit im OP ist zentral für eine sichere Patientenversorgung sowie für die Zufriedenheit des OP-Personals. Mittels einer Kombination von standardisierten Beobachtungen und Befragungen bei einem breiten Spektrum von Routineeingriffen untersuchten wir den Zusammenhang von interprofessioneller Zusammenarbeit und Stresserleben des OP-Personals. Für eine Vielzahl unterschiedlicher Eingriffe wurden professionsspezifische Zusammenhänge festgestellt, die ein vertieftes Verständnis für das Zusammenspiel von gelingender OP-Teamarbeit und Stress in der operativen Routineversorgung über unterschiedliche Fachbereiche ermöglichen.

Die durchschnittlichen Ausprägungen der interprofessionellen Teamarbeit (OTAS-D-Wertungen) lagen für das gesamte Team sowie für die einzelnen OP-Professionen im mittleren, leicht positiven Bereich, mit vergleichsweise geringen Varianzen. Unsere Ergebnisse sind vergleichbar zu ähnlichen, internationalen Untersuchungen: Auch hier liegen die OTAS-Wertungen der intraoperativen Phase im positiven Bereich mit geringeren Schwankungen [11, 36], wobei Hull et al. [11] in einigen Kategorien etwas höhere, Undre et al. [31] aus der Urologie durchgängig höhere Werte berichteten.

Eine Stärke unserer Studie ist die Kombination der Beobachtungen mit den subjektiven Bewertungen des OP-Personals. Ebenfalls konsistent zu ähnlichen Studien im operativen Routinebetrieb rangierten die berichteten Stresslevel im unteren Drittel der Gesamtskala [11]. Für die 3 Subteams der Chirurgie, Pflege und Anästhesie ergaben sich bedeutsame Unterschiede im Stresserleben mit höchsten Werten für das chirurgische Team. Unsere Ergebnisse verdeutlichen, dass sich die OP-Disziplinen in ihrem Stresserleben auch während alltäglicher Eingriffe mit routinierten Anforderungen und Prozeduren unterscheiden [11, 28]. Vergleichbar zu früheren Beobachtungen berichtet das chirurgische Subteam tendenziell mehr Stress als Pflegekräfte und Anästhesie [11, 36].

Bemerkenswert ist, dass keine signifikanten Unterschiede im Stresserleben in Abhängigkeit von der Berufserfahrung gefunden wurden. Post-hoc sind verschiedene Erklärungen möglich: Wir beobachteten Routineeingriffe mit einem insgesamt niedrigeren Stresslevel als kritische Situationen, in denen Berufserfahrung und einhergehende Stressresilienz bedeutsamer sind; die Ergebnisse basieren auf Selbstaussagen; und es sind weitere Einflussfaktoren denkbar, beispielsweise steigt mit der Berufserfahrung auch der Grad der Verantwortung, Führung, Supervision und Aufgabenkomplexität [27].

Wir beobachteten, dass das Stresserleben aller Teammitglieder mit der Dauer des Eingriffs korreliert, am stärksten jedoch für das chirurgische Team. Fortschreitende Operationszeit ist demnach besonders für das chirurgische Subteam ein Stressfaktor, möglicherweise bedingt durch die kontinuierliche Aufmerksamkeitszuwendung, kognitive Belastung, Erschöpfungsrisiken und mangelnde Möglichkeiten der Erholung. Kennedy-Metz et al. konnten diesen Zusammenhang für Bypass-Eingriffe nachweisen [13]. Zudem beanspruchen komplizierte, anforderungsintensive Eingriffe meist mehr Zeit. Gerade bei den verantwortlichen Hauptoperateuren steigen mit der Komplexität und der Dauer des Eingriffs sowohl die körperliche als auch die psychische Belastung [14].

### Zusammenhänge zwischen der Güte der OP-Teamarbeit und dem Stressniveau

Im multivariaten Modell ergaben sich keine Hinweise auf Zusammenhänge zwischen der Teamarbeit des Gesamtteams und dem Stresserleben über alle Befragten. Wie in früheren Untersuchungen zeigte sich, dass sich die 3 OP-Professionen dahingehend unterschieden, wie Teamarbeit das Stresserleben beeinflusst [8, 28]. Wir beobachteten Zusammenhänge zwischen der Teamarbeit und dem Stresserleben der Chirurgie und der Pflege; einerseits jeweils für die Teamarbeit im Gesamten als auch für die Chirurgen für die Einzeldimensionen Zusammenarbeit und Führung. Für die OP-Pflege war gute Teamarbeit – insbesondere Team-Monitoring – mit geringerem Stress assoziiert, was ebenfalls konsistent zu früheren Studien ist [20, 28]. Zwar können keine Aussagen über Kausalzusammenhänge getroffen werden, dennoch wurden so Zusammenhänge zwischen Teamarbeit und dem Stresserleben der Professionen im Routinebetrieb sichtbar.

Entgegen unserer Annahmen zeigen unsere Ergebnisse, dass mit besserer intraoperativer Teamarbeit ein erhöhtes Stressniveau für das chirurgische Team einherging. Dieser Zusammenhang fand sich ebenfalls für die Teamarbeitsdimensionen Zusammenarbeit und Führung. Auch wenn ein Zusammenhang zwischen Stress und „non-technical skills“ wie Teamarbeit und Kommunikation für das chirurgische Subteam bereits in der Literatur berichtet wurde [3, 4, 6, 11], ist die Richtung des Zusammenhangs unerwartet. Post hoc sehen wir verschiedene Erklärungen: Einerseits könnte der Grad an notwendiger Teamarbeit sowie des Stresses durchaus von der technischen Komplexität der Eingriffe abhängen [5]. Komplexe Eingriffe, die mit einer erhöhtem physischen und psychischen Belastung einhergehen, fordern ein erhöhtes Maß an Kommunikation, Koordination, Zusammenarbeit und Führung [14, 34]. Auch zeigen Chirurgen bei hoher Komplexität mehr Führungsverhalten [21] und sind dadurch mehr gefordert. Wir vermuten, dass die beobachtete Teamarbeit daher auch ein Kompensationsmechanismus sein könnte, um stressintensive, komplexe Anforderungen besser zu bewältigen. Die Anforderung, sich nicht nur auf das unmittelbare Operationsgebiet sowie die Zusammenarbeit innerhalb des Subteams zu konzentrieren, sondern sich interdisziplinär auszutauschen und eine Führungsrolle einzunehmen, könnte auch selbst eine genuine Stressquelle für die Chirurgen darstellen. Angesichts beanspruchter mentaler Kapazitäten könnten die Steuerung und Regulation der intraoperativen Teamarbeit auch anstrengend sein. Die Ergebnisse unserer Studie bedürfen daher nicht nur einer sorgfältigen Replikation, es ist auch vertiefte Forschungsarbeit notwendig, beispielsweise um den Einfluss des Schwierigkeitsgrades des Eingriffs von den genuinen Anforderungen für die Steuerung der OP-Teamarbeit abzugrenzen, auch um einzelne Fächer und Eingriffe genauer zu untersuchen und umfänglichere Daten unterschiedlicher Kliniken und Teams zu gewinnen.

### Stärken und Limitationen

Die Studie hat trotz ihres anspruchsvollen Designs sowie des Umfangs an Beobachtungen verschiedene Grenzen. Die Anzahl der 64 Beobachtungen erstreckte sich über eine Vielzahl von Eingriffen und Fächern. Unser Stichprobenumfang ist vergleichbar zu früheren Untersuchungen [12, 27, 36]. *Alle Beobachtungen wurden in OP während elektiver Eingriffe durchgeführt, aber auch hier sind* inhärente Limitationen von Beobachtungen zu beachten, wie Auswahl- und Beobachtereffekte, die gerade bei Teamarbeitserhebungen eher positive Ergebnisse induzieren können. Obwohl wir ein etabliertes Verfahren nutzten, können Limitationen dieser Methode wie ungenügende Berücksichtigung eingespielter oder nonverbaler Koordinationsabläufe und der Grad der Vertrautheit des Teams für das Ergebnis mitverantwortlich sein [13, 15]. Der chirurgisch-technische Schwierigkeitsgrad sowie der Ausbildungsstand und Aufgabenbereich der einzelnen Teammitglieder wurden nicht miteinbezogen. Individuelle Unterschiede einzelner Personen im Umgang mit Stress wurden auch nicht berücksichtigt. Um diese und andere Faktoren zu kontrollieren, würden sich entsprechende Simulationsstudien anbieten. *Als Komplexitätsmaß mit möglichen Auswirkungen auf des Gesamtteam wurde der ASA-Score einbezogen*, jedoch zeigen unsere Ergebnisse, dass dieser nur einen Einfluss auf das Stressempfinden des anästhesiologischen Teams hat. Für die Erfassung des Stresses nutzten wir retrospektive Selbstaussagen mit dem Risiko für Einschätzungsfehler („hindsight bias“). Zwar ist die Inhaltsvalidität der verwendeten Einzelfrage für psychophysischen Stress gewährleistet, zukünftige Erhebungen sollten hier nichtsubjektive Verfahren einbeziehen (als objektivierte intraoperative Stressparameter, wie Augenbewegung, dermale Leitfähigkeit, Cortisol).

## Fazit für die Praxis

- Auch im alltäglichen Routinebetrieb entsteht subjektiv empfundener Stress und ist somit ein Faktor, der immer berücksichtigt werden muss, nicht nur bei sehr komplexen und/oder längeren Eingriffen, wobei
- die verschiedenen Professionen im OP durch unterschiedliche Faktoren in ihrem Stresserleben beeinflusst werden,
- ein allgemeiner Zusammenhang zwischen der Güte der Teamarbeit und dem Stressempfinden, der gleichermaßen für alle OP-Professionen gilt, nicht beobachtet werden konnte, da
- Teamarbeit und Stress in einem so komplexen Zusammenhang stehen, dass pauschale Schlussfolgerungen über Wirkrichtungen (auf Basis dieser Erhebung) nicht möglich sind.

## Supplementary Information




