# Supplementary material for: Teamwork and stress in routine interventions: an observational study of multiprofessional OR teams
Source: Unfallchirurg. 2021 Mar 5;125(2):130–7. [Article in German] doi: 10.1007/s00113-021-00977-w (PMC8813711; doi:10.1007/s00113-021-00977-w)
Supplement: Supplementary file 1 [file 113_2021_977_MOESM1_ESM.pdf]

Electronic Supplementary Material

Tabelle E 1: Liste und Kurzbeschreibung beobachteter Eingriffe

Beob.-Nr.	Schnitt-Zeit	Verschluss-Zeit	Beschreibung Eingriff	ASA-Score	Beobachter	Anwesende (intra-op)	Besonderheiten / Anmerkungen
1	11:45	12:40	VAC-Wechsel mit Materialentfernung	2	BEOB1; BEOB3	3 Chirurgen; Anästhesistin; instrumentierende Pflegekraft, Springer	HIV-Patient (geringe Viruslast); instrumentierende Pflegekraft muss bei Röntgen für ein paar Minuten den Saal verlassen da sie keine Schürze trägt; Wechsel des Anästhesisten (Pausenvertretung)
2	13:50	14:15	Rekonstruktion des Ellenbogens	2	BEOB1; BEOB3	3 Chirurgen; Anästhesist, instrumentierende Pflege, meist 2 Springer; Zuschauer (Famulantin)	Gastarzt spricht nur englisch
3	11:35	14:55	tief-anteriore Rektumresektion mit koloanaler Anastomose (laparoskopisch)	2	BEOB1; BEOB3	3 Chirurgen und 1 PJ‘ler am Tisch; Anästhesist; instrumentierende Pflege, Springer	ca. 12:20 Wechsel Anästhesist (Pausenvertretung)
4	9:07	10:05	Osteosyntheseverfahren durch Schraube (Diagnose: Fraktur des Femurs)	2	BEOB1; BEOB3	Chirurgie: Hauptoperateur, Assistent, PJ’ler am Tisch; Anästhesist; instrumentierende Pflege, (teilweise 2) Springer; Gastarzt als Beobachter	dem Beobachter wird einiges erklärt, hierzu muss sich Hauptoperateur umdrehen; zudem Englisch sprechen

**5**	11:20	12:30	Osteosyntheseverfahren durch Schraube (Diagnose: Fraktur des Femurs)	1	BEOB1; BEOB3	3 Chirurgen; Anästhesist; instrumentierende Pflege, Springer; Gastarzt als Beobachter	Gastarzt als Beobachter im Saal; Hauptoperateur geht um 12:15; Anästhesist wechselt (während Zunähen um 12:25)
**6**	13:25	15:45	Osteosyntheseverfahren durch Platte (Humerusfraktur)	2	BEOB1; BEOB3	2 Chirurgen (zweiter kommt erst kurz nach Schnitt) + PJ'ler am Tisch; Anästhesist; instrumentierende Pflege, Springer	Wechsel des Anästhesisten um 13:45 (gute Übergabe), kommt wieder um 14:25; Springer wechselt 3 mal
**7**	8:50	9:20	Rektum Stimulation bei analer Inkontinenz	2	BEOB2; BEOB3	2 Chirurgen (+ Vertreter von Ausrüstungsfirma, instruiert Chirurgie); Anästhesist instrumentierende Pflege, Springer (+ 2 beobachtende Schüler)	Vertreter Instrumentenfirma (Ausrüstung Schrittmacher) anwesend und spricht mit / instruiert Chirurgen
**8**	11:15	12:10	Rekonstruktion Abfluss der Lebervene; Z.n. Leberoperation (Revisions-OP)	3	BEOB2; BEOB3	2 Chirurgen zu Beginn 1 Anästhesie-Assistenzarzt + 2 Anästhesie-Pfleger (bis 20 Minuten nach OP-Beginn), zeitweilig 1 Anästhesie-OA, zeitweilig 1 Anästhesie-Pflege + 1 PJ'lerin; instrumentierende Pflege, Springer	während OP teilweise Chefarzt anwesend
**9**	13:25	15:21	CrDSS over Venen Stripping	3	BEOB2; BEOB3	2 Chirurgen; Anästhesist; Springer, instrumentierende Pflege + 1 Schüler	Wechsel Anästhesist unter OP; sehr ruhige OP (wenig Unterbrechungen)

**10**	10:40	12:17	Diagnostische Arthroskopie des Handgelenks	2	BEOB1; BEOB3	Chirurg + 1 PJ'ler am Tisch, später 2 Chirurgen; Anästhesistin; instrumentierende Pflege, Springer	zweiter Chirurg kommt im 11:50 hinzu; Wechsel Anästhesie 11:55
**11**	13:30	15:05	Arthroskopie der Schulter (laparoskopisch)	2	BEOB1; BEOB3	2 Chirurgen; Anästhesist; instrumentierende Pflege, Springer; Zuschauer: Gastarzt (englischsprachig); 2 PJ'ler;	Erläuterungen an zusehenden Gastarzt auf Englisch; nicht komplett angeleitete OP: schwierigeren Teil übernimmt OA; instrumentierende Pflegekraft geht kurz vor Ende der Operation (14:55)
**12**	8:45	11:00	Hiatushernie-Fundoplikation (laparoskopisch mit neuer Roboterunterstützung)	2	BEOB2; BEOB3	2 Chirurgen, 2 Vertreter der Roboterfirma Anästhesistin 3 chirurg. Pflege: instr. Pflege + Springer + Helferin (+ teilweise Stützpunktleitung)	neues Equipment (Einsatz eines OP-Roboters mit Kameraarm; Vertreter anwesend)
**13**	12:08	14:28	Splenektomie (offen)	4	BEOB2; BEOB3	2 Chirurgen + 1 PJ'ler nach 30 min; Anästhesie: Oberarzt, Assistenzarzt Instrumentierende Pflege, Springer, Azubi	OP-Instrumente anfänglich nicht da; Patient insgesamt kritischer Zustand
**14**	9:10	9:45	Ileostomaanlage bei Rektumkarzinom (laparoskopisch)	3	BEOB1; BEOB3	2 Chirurgen; Anästhesist + PJ'lerin; instrumentierende Pflege, Springer	Wechsel Anästhesie
**15**	9:03	9:36	Sinus pilonidalis Exzision nach Karydakis	2	BEOB1; BEOB3	1 Chirurg; Anästhesistin, teilweise A-Pflege + A-Pflege-Schülerin instrumentierende Pflege, Springer,	

**16**	13:04	15:10	Zugurtungsosteosynthese an Patella (Patellafraktur)	2	BEOB1; BEOB3	3 Chirurgen + 1 PJter am Tisch; Anästhesistin instrumentierende Pflege, Springer;	14:58 Wechsel instrumentierende Schwester; 15:07 Springer wechselt
**17**	8:35	9:07	Parathyreoidektomie	3	BEOB1; BEOB3	2 Chirurgen (zweiter Chirurg erst ab 08:40); Anästhesistin, teilweise A-Pflege; instrumentierende Pflege, Springer PJ'ler (Zuschauer)	Anästhesist wechselt kurz vor Schnitt, erste Anästhesistin kommt wieder um 08:45 (teilweise zu zweit); Probleme mit Ableitungen
**18**	10:30	11:05	Verschluss einer Hernia inguinalis (Leistenhernie) (laparoskopisch)	2	BEOB1; BEOB3	1 Chirurg; Anästhesie, A-Pflege zu Beginn; instrumentierende Pflege, Springer PJ'ler (Zuschauer)	
**19**	12:40	14:10	Partielle Nephrektomie (Tumor) (laparoskopisch)	4	BEOB1; BEOB3	2 Chirurgen + Austauschstudentin (Zuschauer); Anästhesie: Assistenzarzt, OA bis 12:50, teilweise A-Pflege; instrumentierende Schwester, Springer; PJ'ler (Zuschauer)	Wechsel Springer 13:05; Anästhesist wechselt um 13:55 (OA übernimmt)
**20**	11:25	12:38	Unterschenkel-Amputation	3	BEOB1; BEOB3	Chirurg + 1 PJ'erlin am Tisch; Anästhesist; instrumentierende Pflege, Springer	Saal ist noch nicht frei als Pat. eintrifft, da OP vorher länger dauert als geplant; Pat. bekommt Schmerzkatheter; Anästhesist wechselt um 11:35
**21**	9:04	10:20	arthroskopische Refixation SSP (Diagnose: subtotale Rotatorenmanschettenruptur)	2	BEOB1; BEOB3	2 Chirurgen; Anästhesist + 1 PJ'ler; instrumentierende Pflege, Springer; 2 Zuschauer	Anästhesist wechselt um 09:15; "Rückwechsel" um 09:50; Wechsel Springer um 10:05

**22**	11:42	13:20	Arthroskopie der Schulter (vermutete Rotatorenmanschettenläsion)	2	BEOB1; BEOB3	2 Chirurgen; Anästhesist; instrumentierende Schwester, Springer; Zuschauer: neue Schwester (die später alleine Springer ist);	Springer ist länger nicht da; teilweise neuer, sehr unerfahrener Springer; Anästhesist wechselt um 12:50
**23**	8:58	9:35	Osteosynthese (Radiusfraktur)	2	BEOB1; BEOB3	2 Chirurgen; Anästhesist; instrumentierende Pflege, Springer, Pfleger der Röntgen bedient 1 Zuschauer (unbekannt)	keine Vollnarkose; Anästhesist wechselt um 09:10, kommt wieder um 09:18
**24**	10:40	12:15	Osteosynthese (Fraktur Clavikula)	1	BEOB1; BEOB3	2 Chirurgen + eine Studentin am Tisch; Anästhesist plus teilweise A-Pflege; instrumentierende Pflege, 2 Springer, teilweise MTA der Röntgen bedient; Zuschauer: Student	
**25**	8:40	10:25	vierfache Hernie (laparoskopisch)	2	BEOB1; BEOB3	2 Chirurgen; Anästhesist; instrumentierende Pflege, Springer, Schwesternschülerin (fungiert teilweise alleine als Springer);	Anästhesist wird zeitweilig vertreten; Springer wechselt um 09:15 (kommt 10 Minuten später wieder)
**26**	11:30	13:25	Cholezystektomie, laparoskopisch	2	BEOB1; BEOB3	2 Chirurgen; Anästhesist; instrumentierende Pflege, Springer, teilweise Schwesternschülerin	Patientin stark übergewichtig (BMI ca. 39); Anästhesist wechselt um 12:05 (Pausenvertretung bis 12:45); (erster) Chirurg geht um ca. 13:25

**27**	9:55	13:35	Tumorentfernung, Halspräparation (medulläres Schilddrüsenkarzinom)	3	BEOB1; BEOB3	2 Chirurgen + 1 Praktikant am Tisch; Anästhesist; instrumentierende Pflege, Springer. teilweise 1 weitere sterile Schwester, teilweise 2. Springer (Schwesternschülerin); Zuschauer: 2 PJ'ler;	Anästhesist wechselt um 11:30; zuschauende PJ'ler gehen um 11:55; Eingriff dauert normalerweise um die 6 Stunden; Chirurgen haben aber „straight, konzentriert, hervorragend gearbeitet" --> in kürzerer Zeit geschafft (eigene Aussage Chirurgie)
**28**	15:05	15:34	Resektion dorsales Handgelenks Ganglion	1	BEOB1; BEOB3	1 Chirurg + 1 Gastarzt aus USA am Tisch; Anästhesistin; instrumentierende Pflege, Springer; Zuschauer: 2 PJ'ler	
**29**	8:30	9:50	Hemithyreoektomie + zentrale Halspräparation (Diagnose: suspekter Schilddrüsenknoten)	2	BEOB1; BEOB3	Chirurgen + 1 Student am Tisch; Anästhesistin + PJ'lerin, teilweise A-Pflege; instrumentierende Pflege, teilweise 2 Springer; teilweise 2 Zuschauer (Studenten);	1. Chirurg geht um 09:30
**30**	10:45	11:30	Ileostoma-Rückverlagerung	3	BEOB1; BEOB3	2 Chirurgen + 1 PJ'ler am Tisch; Anästhesist, teilweise A-Pflege; instrumentierende Schwester, 2 Springer (einer davon recht neu);	Anästhesist wechselt um 11:15
**31**	12:35	13:07	Cholezystektomie, laparoskopisch	2	BEOB1; BEOB3	2 Chirurgen + PJ'ler am Tisch; Anästhesist, teilweise A-Pflege instrumentierende Pflege, Springer (teilweise nicht da); Pflege-Schülerin;	Hepatitis-B-Patientin; Schwesternschülerin fungiert als Springer wenn eigentlicher Springer abwesend ist; 1. Chirurg geht um 12:55

**32**	8:55	9:35	Rekonstruktion Kapselbandapparat (mit Schraube); Diagnose: Ellenbogenluxation, knöcherner Ausriss ulnare Sehne	2	BEOB1; BEOB3	2 Chirurgen + 1 PJ'ler am Tisch; Anästhesist; instrumentierende Pflege, Springer	
**33**	10:35	12:03	Plattenosteosynthese, distaler Radius	1	BEOB1; BEOB3	2 Chirurgen; Anästhesist; instrumentierende Pflege, (teilweise 2) Springer; Zuschauer: 1 PJ'ler;	ein Chirurg übernimmt zeitweise den Part der instrumentierenden Schwester (Grund unklar); Bohrer funktioniert nicht richtig
**34**	13:15	14:25	Arthroskopie Knie + OATS-Plastik	3	BEOB1; BEOB3	2 Chirurgen; Anästhesist; instrumentierende Pflege, 2 Springer; Zuschauer: 1 PJ'ler (später ab ca. 13:50 am Tisch)	Anästhesist wechselt um 14:10
**35**	9:45	10:45	Leistenhernie (TAPP) (laparoskopisch)	2	BEOB1; BEOB3	2 Chirurgen; Anästhesistin; instrumentierende Pflege, Springer; teilweise Schwesternschülerin; Zuschauer: Studentin	
**36**	12:15	12:50	Perkutane Verschraubung Herbertschraube (Scaphoidfraktur)	1	BEOB1; BEOB3	2 Chirurgen; Anästhesist; instrumentierende Pflege, Springer; 4 Zuschauer (Studenten; Gäste)	
**37**	9:30	13:27	mikroskopischer Mitralklappenersatz (MKE)	3	BEOB1; BEOB3	2 Chirurgen + 1 Studentin am Tisch; Anästhesist; instrumentierende Pflege, Springer, Schwesternschülerin; Kardiotechniker; diverse Zuschauer (2	allgemein immer mal wieder wechselnde Zuschauer; assistierende Chirurgin geht um 13:15

						davon englischsprachig); später ein Vertreter einer Firma (Instrumentenherstellung)	
**38**	8:45	9:30	PE bei Magenkarzinom (Magenvorderfläche) (laparoskopisch)	3	BEOB2; BEOB3	2 Chirurgen; Anästhesist; instrumentierende Pflege, Springer + Helfer Pflege	
**39**	13:05	12:46	Bauchlückenverschluss - BLV mit Netz, offen (Narbenhernie)	3	BEOB2; BEOB3	2 Chirurgen; Anästhesistin, A-Pflege; Instrumentierende Pflege, Springer + Schülerin	Springer wechselt unter OP (Pausenvertretung)
**40**	9:53	11:32	Y-Stent-Graft (Diagnose: BAA)	4	BEOB1; BEOB3	2 Chirurgen; Anästhesist; instrumentierende Pflege, Springer, MTA am Röntgengerät	keine Vollnarkose; Kommunikation mit dem Patienten notwendig, da der beim Einsetzen des STENTs die Luft anhalten muss; Patient spricht aber kein Deutsch --> MTA am Röntgen übersetzt / übernimmt Anweisungen auf Russisch; Springer wechselt 10:50, "Rückwechsel" um 11:10
**41**	12:47	14:58	TEA + Patch linke AFC (Thrombektomie)	3	BEOB1; BEOB3	2 Chirurgen, ab 13:20 Studentin am Tisch; Anästhesist instrumentierende Pflege, Springer	Pausenvertretung Springer von 13:10 bis 13:40; OA-C (1. Chirurg) geht um 14:15

**42**	8:30	9:45	Verschluss Leistenhernie (Netz), laparoskopisch	2	BEOB2; BEOB3	2 Chirurgen (OA 20 min nach Schnitt); Anästhesist + PJ'ler; Intrumentierende Pflege, Springer	OA Chir kommt dazu, recht lebendig dann; Assistenz-Arzt operiert die ganze Zeit unter Anleitung / Supervision; OA verlässt den OP nachdem das Netz angenäht ist
**43**	12:10	12:56	Cholezystektomie, laparoskopisch	2	BEOB2; BEOB3	2 Chirurgen; Anästhesist + zeitweise A-Pflege; Instrumentierende Pflege, Springer	hektische Phase nachdem Patient in OP war bis zum Start der OP, unter OP recht ruhig
**44**	9:58	10:45	Osteosyntheseverfahren durch (Medartis)Platte; Handgelenk	2	BEOB1; BEOB3	Chirurg; Anästhesistin; instrumentierende Pflege, Springer, teilweise MTA (Röntgen)	keine Vollnarkose; Anästhesistin wechselt; Springer spricht nicht gut deutsch und ist neu
**45**	11:29	12:05	Metallentfernung (Entfernung Platte Schulter)	1	BEOB1; BEOB3	Chirurg + ab 11:40 PJ'ler am Tisch; Anästhesistin; Instrumentierende Pflege, Springer	Springer spricht nicht gut Deutsch und ist neu
**46**	13:01	13:25	Restgastrektomie	2	BEOB2; BEOB3	2 Chirurgen (nach 60 min + 1 PJ'tler); Anästhesie + A-Pflege; Instrumentierende Pflege, 2 Springer	chirug. Pflege kurz pausiert unter OP (von Springer abgelöst)
**47**	10:45	13:25	ASK Meniskus Teilresektion, Zystenauffüllung, RIA-Spongiosegewinnung rechts, evtl. OATS-Plastik (laparoskopisch)	2	BEOB1; BEOB3	2 Chirurgen + englischsprechender Gastarzt am Tisch; Anästhesist; instrumentierende Pflege, Springer; teilweise ein Zuschauer, zeitweise 1 Fotograf	Anästhesie wechselt, wenden recht neue Methode an, wird viel drüber geredet; erster Chirurg verlässt Tisch um 13:15; Zweitweise werden Fotos gemacht

**48**	8:40	11:00	Bauchwandhernie; Netzeinlage (offen)	3	BEOB1; BEOB3	2 Chirurgen; Anästhesist; instrumentierende Schwester, Springer + teilweise Schwesternschülerin	HIV-Patient (geringe Viruslast); Chirurgen kommen recht spät; Anästhesist wechselt um 09:20 (Pausenvertretung bis 09:45)
**49**	11:50	13:35	offene Hemicolektomie mit Anastomose	2	BEOB1; BEOB3	3 Chirurgen am Tisch; Anästhesist; instrumentierende Pflege, Springer; kurzzeitig als Zuschauer: Studentin	Springer wechselt; chirurgischer OA geht um 12:55
**50**	14:03	14:23	transperitoneale Nabelhernie, Netzeinsatz, laparoskopisch, IPOM	2	BEOB1; BEOB3	2 Chirurgen; Anästhesist; instrumentierende Pflege, Springer	
**51**	9:25	10:05	Ileostoma-Rückverlagerung	3	BEOB1; BEOB3	2 Chirurgen; Anästhesist; instrumentierende Pflege, Springer; teilweise zweiter Springer und Besucher	1. Chirurg geht um 10:00
**52**	9:02	10:15	Osteosynthese mittels Philosplatte (Humeruskopffraktur)	2	BEOB1; BEOB3	3 Chirurgen am Tisch (davon ein englischsprachiger Gastarzt; Anästhesistin; instrumentierende Pflege, Springer	Hauptoperateur erklärt Gastarzt viel auf Englisch; Springer recht unerfahren; Anästhesist wechselt um 9:20
**53**	11:35	13:00	Osteosynthese durch Philos-Platte, Humerusfraktur	2	BEOB1; BEOB3	2 Chirurgen; Anästhesist; instrumentierende Pflege, Springer; teilweise Zuschauer (Gast-Student)	instrumentierende Pflege wechselt um 12:20; Springer wird instrumentierend, neuer Springer kommt; ein Chirurg geht um 12:48

**54**	9:00	9:53	Verschluss abdomialer Hernien (offen)	3	BEOB1; BEOB3	2 Chirurgen + eine PJ'lerin am Tisch; Anästhesist + eine Studentin instrumentierende Pflege, Springer;	Patient HIV-positiv
**55**	11:19	12:55	Osteosynthese mittels Philosplatte, Schulter	3	BEOB1; BEOB3	2 Chirurgen; Anästhesie; instrumentierende Pflege, Springer; Zuschauer: englisch sprechender Gaststudent	Erklärungen an Gast auf Englisch; Anästhesistin wechselt bei Beginn OP, kommt zurück um ca. 11:40; Springer wechselt; erster Chirurg verlässt Tisch um 12:45
**56**	9:47	12:50	Recrossektomie Leiste, SA + Perforansligatur (Diagnose: Rezidivvarikosis)	2	BEOB1; BEOB3	2 Chirurgen + Studentin am Tisch; Anästhesist + PJ'ler; instrumentierende Pflege, Springer	Anästhesist wechselt um 11:30 (Pausenvertretung), kommt wieder um 12:00; erster Chirurg verlässt Tisch im 12:45
**57**	14:25	16:45	arthroskopisch assistierte BK Auffüllung Knie; Spongiosaentnahme ipsilateral femur (Diagnose: vordere Kreuzbandinsuffizienz)	2	BEOB1; BEOB3	2 Chirurgen; Anästhesie; instrumentierende Pflege, teilweise 2 Springer	Wechsel Anästhesie um 15:00; instrumentierende Pflege wechselt um 15:40, zweite instrumentierende Schwester besseres Monitoring; Wechsel auch bei Springer
**58**	8:50	9:35	diagnostische Laparoskopie; (Z.n. Pankreaslinksresektion bei Pankreaskarzinom)	2	BEOB1; BEOB3	2 Chirurgen; Anästhesist; instrumentierende Schwester, Springer	Anästhesist wird am Anfang der OP mal kurz vertreten
**59**	12:15	13:15	VATS Hämatomausräumumg bei Hämatothorax (laparoskopisch)	3	BEOB1; BEOB3	2 Chirurgen; Anästhesist + A-Pflege, instrumentierende Pflege, Springer; teilweise Schwesternschülerin	

60	9:50	10:42	Leistenhernie; TAPP (laparoskopisch)	2	BEOB1; BEOB3	2 Chirurgen; Anästhesist + 1, teilweise 2 PJ'ler / Famulant; instrumentierende Schwester, Springer	bei Ausleitung keine A-Pflege da, nur Famulant
61	9:18	12:09	Leberresektomie (geplant; jedoch unter OP entschieden, Leber partiell zu entfernen)	3	BEOB2; BEOB3	2 Chirurgen + 1PJ'ler am Tisch; Anästhesist + A-Pflege; Instrumentierende Pflege, Springer	(Zertifizierung angekündigt); Haupt-Operateur erklärt nach OP, es wäre eine anstrengende OP gewesen und: „sehr schwieriger Eingriff"; Planänderung während des Eingriffs (Leber wird halbiert)
62	8:29	9:10	Leistenhernie rechts	2	BEOB2; BEOB3	2 Chirurgen; Anästhesistin + A-Pflege; Instrumentierende Pflege, Springer	Beginn recht hektisch, da Operateur da und Patient noch nicht fertig; sehr schneller, einfacher Eingriff
63	9:57	11:56	Hemikolektomie rechts	3	BEOB2; BEOB3	Chirurg + 1 PJ'ler am Tisch, nach 60 min 2. PF'ler; Anästhesistin; Instrumentierende Pflege, Springer	Time-out nebenher beim Abwaschen; ab Mitte der OP zwei PJ'ler anwesend
64	8:55	11:04	Hiatus Hernie (OP laparoskopische FPL + Hiatusplastik)	2	BEOB2; BEOB3	2 Chirurgen (zweiter Chirurg nach 20 min) + PJ'ler am Tisch; Anästhesist; Instrumentierende Pflege, Springer anfänglich 2 Zuschauer	nach ca. 50 min angeleitete OP (Chirurg übergibt an Ass und supervisiert); letzte 30 min Endoskopie (zur Kontrolle)
